# Virtual-Reality Simulator System for Double Interventional Cardiac Catheterization Using Fractional-Order Vascular Access Tracker and Haptic Force Producer

**DOI:** 10.1155/2015/697569

**Published:** 2015-06-14

**Authors:** Guan-Chun Chen, Chia-Hung Lin, Chien-Ming Li, Kai-Sheng Hsieh, Yi-Chun Du, Tainsong Chen

**Affiliations:** ^1^Department of Biomedical Engineering, National Cheng Kung University, Tainan City 70101, Taiwan; ^2^Department of Electrical Engineering, Kao Yuan University, Kaohsiung City 82151, Taiwan; ^3^Division of Infectious Diseases, Department of Medicine of Chi Mei Medical Center, Tainan City 71004, Taiwan; ^4^Kaohsiung Chang Gung Memorial Hospital, Kaohsiung City 833, Taiwan; ^5^Department of Electrical Engineering, Southern Taiwan University of Science and Technology, Tainan City 71005, Taiwan

## Abstract

This study proposes virtual-reality (VR) simulator system for double interventional cardiac catheterization (ICC) using fractional-order vascular access tracker and haptic force producer. An endoscope or a catheter for diagnosis and surgery of cardiovascular disease has been commonly used in minimally invasive surgery. It needs specific skills and experiences for young surgeons or postgraduate year (PGY) students to operate a Berman catheter and a pigtail catheter in the inside of the human body and requires avoiding damaging vessels. To improve the training in inserting catheters, a double-catheter mechanism is designed for the ICC procedures. A fractional-order vascular access tracker is used to trace the senior surgeons' consoled trajectories and transmit the frictional feedback and visual feedback during the insertion of catheters. Based on the clinical feeling through the aortic arch, vein into the ventricle, or tortuous blood vessels, haptic force producer is used to mock the elasticity of the vessel wall using voice coil motors (VCMs). The VR establishment with surgeons' consoled vessel trajectories and hand feeling is achieved, and the experimental results show the effectiveness for the double ICC procedures.

## 1. Introduction

Cardiovascular diseases (CVD), such as coronary artery disease (CAD) and cerebrovascular disease (or stroke), are major risk factors for mortality and morbidity in Taiwan. They were the second and third most leading causes of death in 2013. CAD is a complex chronic inflammatory disease characterized by atherogenesis that would remodel and narrow coronary arteries. The risk factors for CAD, CVD, and peripheral arterial disease are nearly identical, which include hypertension, diabetes mellitus, dyslipidemia, and cigarette smoking. Angiography of the coronary artery is an essential methodology for the systemic evaluation of the coronary tree followed by intervening angioplasty or valvuloplasty. However, particularly in fragile, narrow, and tortuous blood vessel, it is difficult for young surgeons/postgraduate year (PGY) students to operate double catheters. Surgical training needs physical practices on patients under the supervision of senior faculty in teaching institutions. These young surgeons require a simulated learning environment or need more skills/experiences to operate double catheters. Therefore, a virtual-reality (VR) simulator system is necessary for such intended applications.

**Figure 1:**
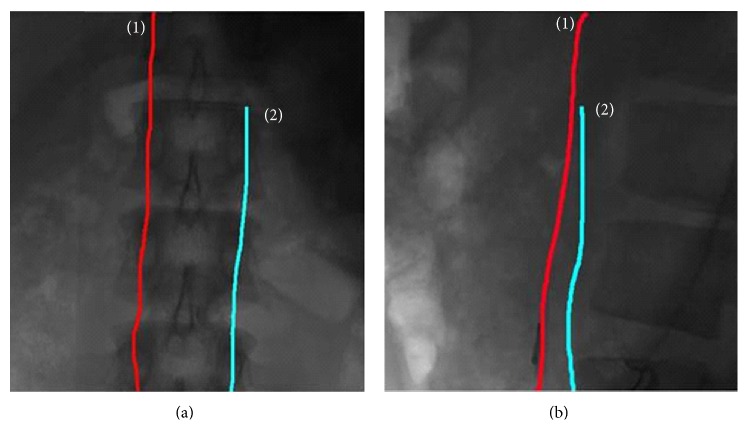
Double interventional cardiac catheterization (ICC): (a) frontal image, (b) lateral image. Note: (1) Berman catheter, (2) pigtail catheter.

Currently, minimally invasive surgery (MIS) is applied in the diagnosis and surgery using an endoscope or a catheter for neurosurgery and for endovascular diseases, such as aneurysm, atrial septal defect (ASD), embolization, and cerebral aneurysm [1–3]. To perform a double ICC procedure for ASD surgery, these surgeries require a Berman catheter inserted from the right femoral vein into the ventricle and a pigtail catheter inserted from the left femoral artery through the right ventricle into the aortic arch, as shown in Figure 1. The first one can insert a sizing balloon catheter and then inject the contrast medium into the heart. It is designed to measure cardiovascular structure and the diameter of an ASD. Surgeries can choose a proper occluder with the X-ray image (radiography) or ultrasonography. The second catheter is used to measure the blood pressure in the left atrium and make sure of the anatomic locations, such as left atrium or left ventricle. The advantages of MIS include small incision, short hospital stay, and short recovery time [4].

In this context, previous literature on current products [4–8] has described the application of robot-assisted catheter system and three-dimensional (3D) computer-assisted technologies for both diagnosis and surgery training. The ANGIO mentor (OKB Medical) is a VR endovascular surgical training simulator, which is used to train inexperienced PGY students to perform ICC and intravascular neurosurgery [4, 5]. However, it lacks the haptic force and force feedback to the surgeons. Sensei robotic navigation system (Hansen Medical) controls catheter navigation by steerable sheaths [6]. It provides the surgeons with multiple degrees of freedom and force detection. Catheter navigation is achieved using 3D computer-assisted systems. The robotic arm obeys the commands from the computer in the control room and then applies tension to the wires embedded within the steerable sheaths. However, force detection at the distal tip is very difficult. NeuRobot uses telesurgery with a microcatheter and an active guidewire for less invasive and telecontrolled neurosurgery [7, 8]. A microcatheter has two bending degrees of freedom and is made using ionic conducting polymer film actuator. Its master-slave microscopic manipulator system has a rigid endoscope and three robotic arms to perform the neurosurgery, while observing on a 3D monitor.

The above-mentioned robotic catheter systems have force feedback and visual feedback with a user interface for monitoring the contact information between a catheter and a blood vessel for ICC. Therefore, a mechanism is needed to measure the operating force on the catheter that can be transmitted to the master manipulator to produce haptic force feedback to the surgeons. In the literature [7, 9–11], force sensors, proportional-integral-differential (PID) controller, and fuzzy control methods have been applied to produce resistance force and haptic force feedback. However, the VR simulator system needs to design a force-sensing system to measure the contact force on the vessel wall. In addition, fluid controller is employed to adjust the blood pressure, and extra sensors are used to monitor blood pressure. In order to realize the force feedback at a narrow and tortuous blood vessel, fractional-order vascular access tracker and haptic force producer are proposed to achieve the steering position and clinical feeling force. Simulation studies [12–14] have confirmed the application of fractional-order models to mock the dynamic responses on vascular wall, viscoelastic mechanical responses, blood flow, and aorta segment parameter calibrations, which provide a flexible model to fitting biological behaviors. A fractional-order tracker is designed using the Sprott chaotic system [15–17], consisting of a master system (MS) and a slave system (SS). A coupling master-slave variable is used, so that the response of the SS can automatically track the response of the MS, intending to trace the “steering position” and transmit the “visual feedback” during ICC procedure. Self-synchronized tracking locates the specific minimum dynamic errors for electromechanical system, such as step motor and voice coil motor (VCM) [18, 19], by controlling the duty ratios of a buck-boost converter (BBC) under step-up and step-down input voltages, which allows realizing the “friction forces” and “haptic forces.”

The remainder of this paper is organized as follows: Section 2 describes the VR simulator system and the experimental setup.  Section 3 addresses the methodology description, including fractional-order tracker and haptic force realization. In Sections 4 and 5, experimental results and conclusions provide feasibility tests to validate the proposed VR simulator system for ICC procedure.

## 2. Virtual-Reality (VR) Simulator System

A VR simulator is the use of visual feedback and an e-learning environment to create, impart, practice, and check senior surgeons' experiences using interactive scenarios to reflect surgery situations. Some e-learning instructions include the contrast medium injection, angioplasty operation, graft-stent implantation, and fluoroscopy (iodine-based contrast) with digital subtraction angiography. After instructions, young surgeons or PGY students need about 30 min to complete the guidewire manipulation and fluoroscopy examinations. In ASD surgery, it is important to enhance the safety operation, while avoiding damaging the blood vessel, such as aortic arch and right femoral vein into the ventricle. Therefore, establishing the force and visual feedback between a catheter and vessel wall is the main issue in this study. Based on the clinical feeling, the proposed VR simulator system comprises three functional components that include hardware mechanism, software controller, and digital image processing, which can be described as follows:

- Hardware mechanism: two VCMs (linear VCMs, ±20 VDC), two step motors (5 VDC, 360°), and four encoders (5 VDC, maximum frequency 100 kHz).
- Software controller: fractional-order tracker and haptic force producer.
- Digital image processing: image enhancement process, image binarization, edge detection, and femoral arterial/venous vessel location.

**Figure 2:**
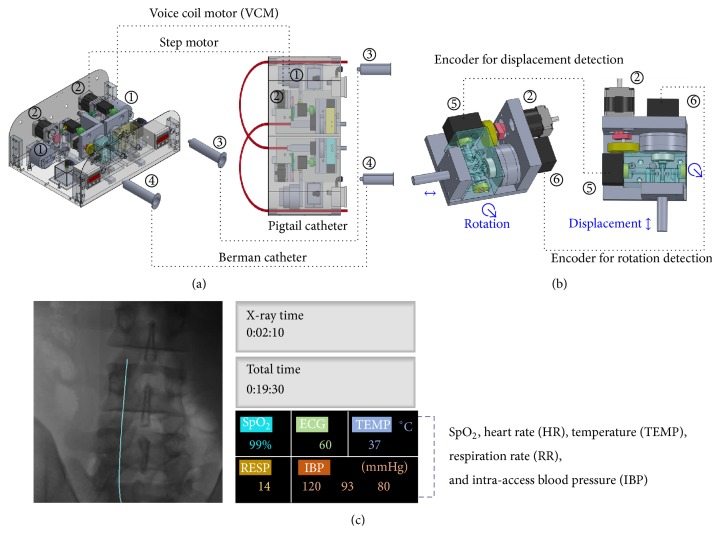
VR simulator system: (a) haptic force and friction force producer for force feedback, (b) rotation and displacement detector for visual feedback, and (c) human-machine interface.

The mechanism of double ICC is shown in Figure 2, which consists of haptic force and friction force producer for force feedback, rotation and displacement detector for visual feedback, and double catheters. The double-catheter mechanism was designed to mock pigtail (symbol: *③*) and Berman (symbol: *④*) catheter operations for ASD VR surgery. The VCMs (symbol: *①*) can mock the haptic forces, while a Berman catheter or a pigtail catheter collides with the vessel wall. In addition, the step motors (symbol: *②*) can mock the friction forces, while the catheters are moving in forward and backward motions or rotating. These motions are detected by 2 encoders for rotation detection and 2 encoders for displacement detection (symbol: *⑤*).

**Figure 3:**
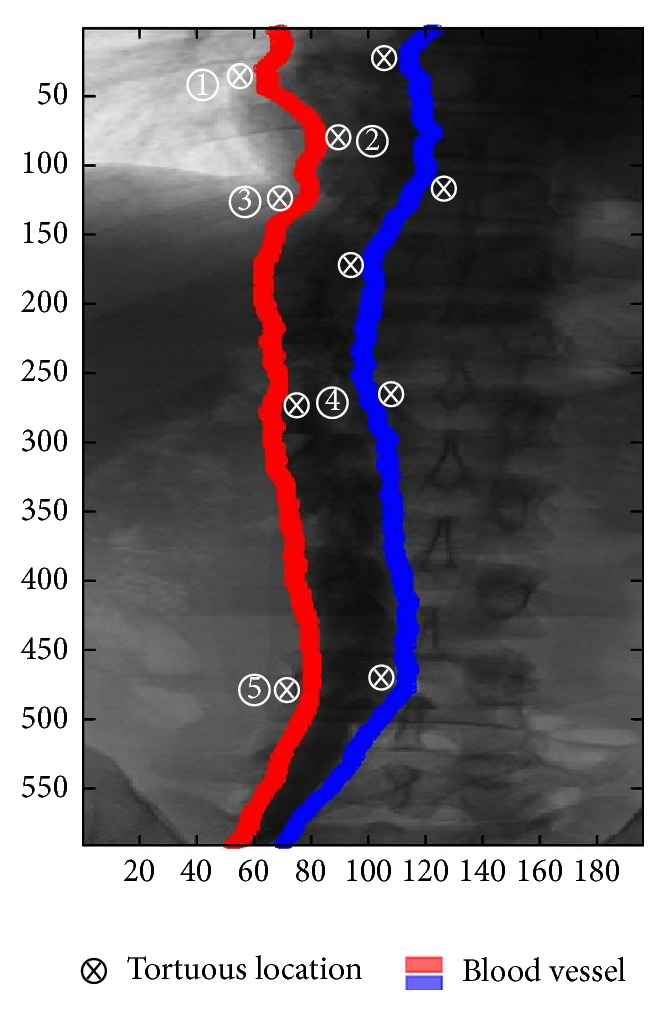
X-ray image, the frontal view of human body in *x*-*y* plane, vessel trajectories as red color and blue color.

As shown in Figure 2(b), two encoders are employed to measure the catheters' displacement and rotation angle, respectively, and then transmit control signal to display the X-ray images on the monitor screen. Displacement measurement calibration is 60 cm (symbol +: forward motion from 0 cm to 60 cm; symbol −: backward motion from 60 cm to 0 cm), and rotation measurement calibration is ±360° (symbol +: clockwise; symbol −: counterclockwise). Every 5° is used to count the catheter rotating motion. An X-ray image is a window of size 512 × 512 pixels, maximum 256 gray scales, and each pixel is 0.367 mm, as shown in Figure 3. Therefore, each catheter displacement and encoder count is the process in which the catheter moves 0.367 mm per encoder scale. This could convert the catheter displacement to the real position of a human body on the monitor screen. The operator can observe and evaluate the safe range, danger warning range, and the dangerous range at any moment, which can be used to monitor the force changes by which a catheter contacts the vessel wall. In addition to the force feedback, the visual feedback control signal can be also transmitted to the young surgeons/PGY students during surgeries. Software controllers, including fractional-order tracker and haptic force producer, are used to automatically produce the friction and haptic forces. A detailed description of the methodology is addressed in Section 3.

## 3. Methodology Description

### 3.1. Digital Image Processing

Digital image processing comprises image enhancement process, image binarization, edge detection, and femoral arterial/venous vessel location. By feeding the digital images into the computer, as shown in Figure 3, the image enhancement process is used to modify the gray-scale values using an intensity transformation function to adjust the contrast between certain intensity values [20–24]. Intensity transformation uses a nonlinear mapping function to enhance the image detail of the original image [24]:

(1) $$\begin{array}{c} g\left(\;r,c\;\right)=\frac{Mk}{\sigma \left(r,c\right)}\left[\;f\left(\;r,c\;\right)-M\left(\;r,c\;\right)\;\right]+M\left(\;r,c\;\right), \end{array}$$

where pixel *g*(*r*, *c*) is individually processed, *M* is the mean of all of the gray-level values, *σ*(*r*, *c*) is the gray-level variance of each 3 × 3 detection mask, *M*(*r*, *c*) is the mean gray-level value for each detection mask, and *k* is a parameter, 0 < *k* < 1. Image edges are boundaries between different gradients in an image. For image segmentation, a first-order derivative is used to identify the discontinuities in intensity from one pixel to another. For the coordinates (*x*_*i*_, *y*_*i*_), *i* = 1,2,…, *n*, the gradient of its coordinate is through a two-dimensional (2D) column vector that can be defined as

(2) ∇2gx,yGxGy=∂2gx,y∂x2∂2gx,y∂y2T,∇gx,yGx2+Gy2≈Gx+Gy.

Using *G*_*x*_ and *G*_*y*_, we can convolve the image to get the gradient. These gradients can be implemented using a 3 × 3 detection mask. First-order/second-order operators and fractional differential filters include Sobel operator, Robert operator, Laplace operator, and fractional differential gradient masks [14, 23, 24]. The edge detection estimator is used to identify the trajectory of the change in image intensity using a 3 × 3 Laplace operator mask. Hence, the femoral arterial/venous vessels can be located. Then a 512 × 512 image is binarized using a threshold value, *ξ*, representing a binary value at the *r*th row and the *c*th column, as

(3) $$\begin{array}{c} b\left(\;r,c\;\right)=\left\{\begin{array}{cc} 1, & g\left(r,c\right)\geq \xi \\ 0, & g\left(r,c\right)<\xi . \end{array}\right. \end{array}$$

**Figure 4:**
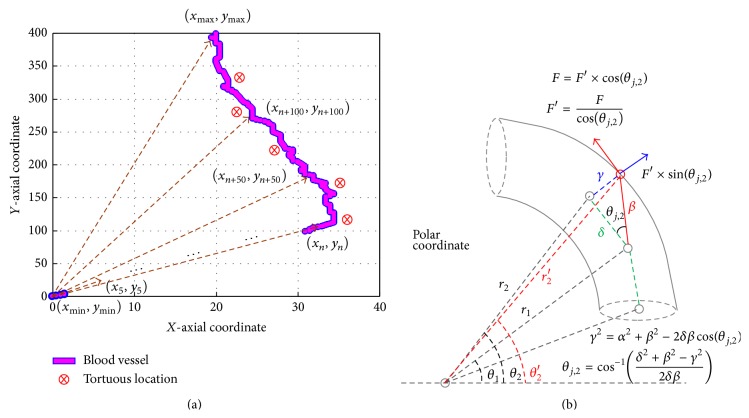
(a) Image coordinates of blood vessel, (b) image polar coordinates of tortuous blood vessel.

Finally, the coordinates of femoral arterial/venous vessels can be represented by red color and blue color in *x*-*y* plane, respectively, as shown in Figure 3. For a 2D X-ray image, Cartesian coordinates convert *x*_*i*_ = *r*_*i*_ × cos(*θ*_*i*_) and *y*_*i*_ = *r*_*i*_ × sin(*θ*_*i*_) to polar coordinates *r*_*i*_ and *θ*_*i*_ with *r*_*i*_ ≥ 0 and *θ*_*i*_ in the interval (−*π*, +*π*], as shown in Figure 4.

### 3.2. Fractional-Order Tracker for Locating the Corner of Tortuous Blood Vessel

**Figure 5:**
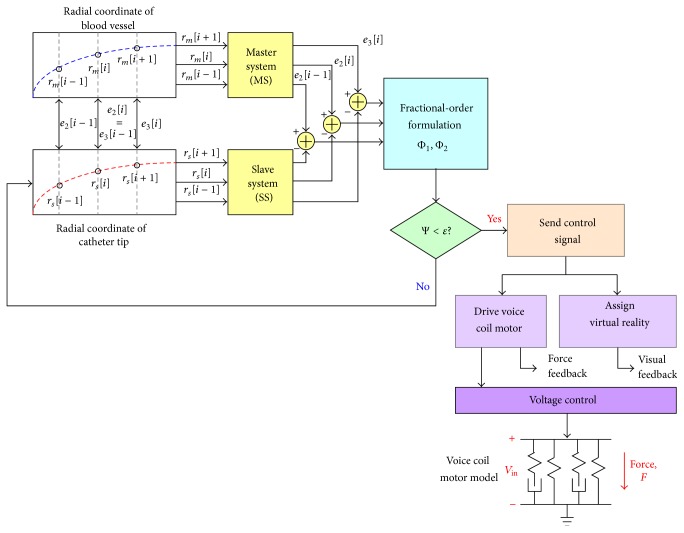
Fractional-order trajectory tracker and force feedback driver.

In order to mock the situation of the intravascular catheter inside a blood vessel, the VR simulator system needs to provide the force feedback to a surgeon/trainer at the corner of the tortuous blood vessel, as shown in Figure 4(b). We propose an access tracker to locate the specific point, while VR simulator system can produce contact force information between the catheter and the blood vessel. Then, VCM is employed to realize haptic force with the mechanical controller and feeds it back to the operator/trainer. Therefore, the trajectory tracker, using the self-synchronization error based on nonlinear tracking model, is used to locate the image polar coordinates of tortuous blood vessel, as shown in Figure 5.

A Sprott system, which consists of an MS as a reference and an SS, is used to design a fractional-order tracker to track the trajectory of the blood vessel and locate the coordinate point of the tortuous blood vessel, as shown in Figure 5, and its general model is defined elsewhere [15–17]:

(4) r˙1mr˙2mr˙3mAr1mr2mr3m+002sign⁡r1m,A=010001−1.2−b−a

shown in Figure 5. Its general model is defined by [15–17]

(5) $$\begin{array}{c} \left[\begin{array}{c} {\dot{r}}_{1s}\\ {\dot{r}}_{2s}\\ {\dot{r}}_{3s} \end{array}\right]=A\left[\begin{array}{c} r_{1s}\\ r_{2s}\\ r_{3s} \end{array}\right]+\left[\begin{array}{c} 0\\ 0\\ 2\mathrm{sign}\left(r_{1s}\right) \end{array}\right], \end{array}$$

where *R*_*m*_ = [*r*_1*m*_, *r*_2*m*_, *r*_3*m*_]^*T*^ are the radial coordinates of the blood vessel, which are determined by a blood vessel extraction algorithm; *R*_*s*_ = [*r*_1*s*_, *r*_2*s*_, *r*_3*s*_]^*T*^ are radial coordinates of the catheter tip and sign is the sign function. If the error variables are defined as *e*_1_ = (*r*_1*m*_ − *r*_1*s*_), *e*_2_ = (*r*_2*m*_ − *r*_2*s*_), *e*_3_ = (*r*_3*m*_ − *r*_3*s*_), and *e* = [*e*_1_, *e*_2_, *e*_3_]^*T*^, by subtracting (5) from (4), the first-order dynamic error system becomes

(6) $$\begin{array}{c} \dot{e}=Ae+2\left[\;\mathrm{sign}\left(\;r_{1m}\;\right)-\mathrm{sign}\left(\;r_{1s}\;\right)\;\right]. \end{array}$$

If both *r*_1*m*_ and *r*_1*s*_ ≥ 0 or *r*_1*m*_ and *r*_1*s*_ < 0, then [sign⁡(*r*_1*m*_) − sign⁡(*r*_1*s*_)] = 0, so a linear system is derived by decoupling variable *e*_1_ from (3), which becomes subjected to system parameters, *a* > 0 and *b* > 0 [15–17]. Consider

(7) $$\begin{array}{c} \left[\begin{array}{c} {\dot{e}}_{2}\\ {\dot{e}}_{3} \end{array}\right]=\left[\begin{array}{cc} 0 & 1\\ -b & -a \end{array}\right]\left[\begin{array}{c} e_{2}\\ e_{3} \end{array}\right]=\tilde{A}\tilde{e} \end{array}$$

subject to system parameters, *a* > 0 and *b* > 0 [15–17].

According to the Grünwald-Letnikov fractional approximation, a general fractional-order differentiation formulation can be expressed as [17, 25]

(8) $$\begin{array}{c} \frac{d^{\alpha }e\left(t\right)}{dt^{\alpha }}=\underset{\Delta t\to 0}{\lim}\frac{e\left(t\right)-\alpha e\left(t-t_{0}\right)}{{\left(t-\left(t-t_{0}\right)\right)}^{\alpha }}\approx \frac{e\left(t\right)-\alpha e\left(t-t_{0}\right)}{{\left(\Delta t\right)}^{\alpha }}. \end{array}$$

If the parameter 0 < *α* < 1 and (8) define the fractional rate of change of the function *e*(*t*), the fractional-order error dynamics of (7) can be expressed as

(9) Dtαe2Dtαe3≈1ΔtαA~e2te3t+0−αΔtααbΔtααaΔtαe2t−t0e3t−t0,

where (*t* − *t*_0_) and *t* points are geometric approximations of the *α*th derivative and the product of the slope between the curves and (Δ*t*)^*α*^ is loosely geometric interpretation of a part of the fractional derivative or fractional rate.

In this study, the fractional-order tracker is used to track the specific points that are the corners of a blood vessel. The radial coordinates of the catheter tip are referred to as *r*_*s*_: discrete variables, *r*_2*s*_ = *r*_*s*_[*i*] and *r*_3*s*_ = *r*_*s*_[*i* + 1], and those from the blood vessel are referred to as *r*_*m*_: discrete variables, *r*_2*m*_ = *r*_*m*_[*i*] and *r*_3*m*_ = *r*_*m*_[*i* + 1], *i* ∈ [1, *n* − 1]. The fractional time, Δ*t*, is the number of radial coordinates, Δ*t* = (*i* + 1) − *i*, *i* ∈ [1, *n* − 1]. Therefore, the fractional-order dynamic system (9) can be modified as discrete fractional-order formulation [17]:

(10) Φ1i=1Δtαe3i−αe3i−1,

(11) Φ2i=−be2i+αe2i−1Δtα+−ae3i+αe3i−1Δtα,

(12) Ψ=Φ1i2+Φ2i2,i=1,2,3,…,n−1,

where error variables are normalized by the maximum radial coordinate, max(*r*_*s*_[*i*]), as

**Figure 6:**
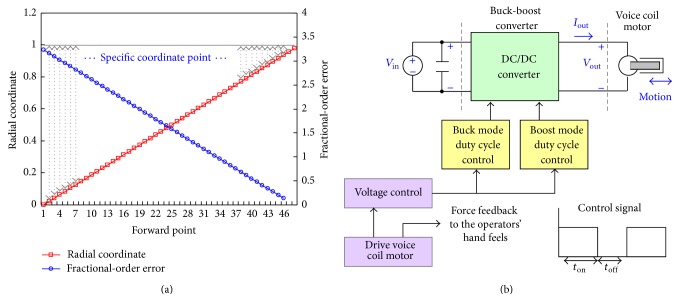
(a) Fractional-order dynamic errors versus catheter tip's displacements (each forward step is 0.367 mm), (b) haptic force producer.

(13) e2ie3i−1=rmi−rsimax⁡rsi,

(14) e2i−1rmi−1−rsi−1max⁡rsi,

(15) e3irmi+1−rsi+1max⁡rsi,

and the initial values are *r*_*m*_[0] = *r*_*s*_[0] = 0. The fractional-order dynamic error, Ψ, is calculated using (12), while Ψ < *ε* means the catheter tip arrives at the specific coordinate point. In this study, the encoder counts the catheter displacement (0.367 mm per encoder scale) and locates the coordinate points on the monitor screen. When fractional-order tracker reaches the specific coordinate points (through the tortuous blood vessel or stenotic segment), then force feedback driver is used to control the VCM with the input voltage regulations. Then, the haptic forces can be produced. With the system parameters, *a* = 1 > 0 and *b* = 0.5 > 0, and *α* = 0.9, the fractional-order dynamic errors versus catheter tip's displacements are shown in Figure 6(a), where each forward step is 0.367 mm.

### 3.3. Haptic Force Realization Using Voice Coil Motor (VCM)

The VR simulator system for ASD or vascular interventional surgery consists of a master-slave manipulator [7, 9, 10]. On the master system, an operator or a trainer can move a guidewire along the axial direction (forward or backward) and radial direction, while the control signal is transmitted to the slave system. During catheterization, as if the operator inserts or rotates a catheter inside the blood vessel, the VR simulator system produces the visual feedback and force feedback [4–7] that are transmitted to the operator in real time. Therefore, when the catheter contacts the blood vessel wall, the force feedback and the operation situation can be transmitted to the operator's hand (feel) and the monitor, respectively.

The catheter can move forward or backward in real-time surgery; it rotates in order to go through the tortuous blood vessel or stenotic segment. In this study, a step motor is used to drive the friction in forward and backward motions, and a VCM actuator is used to drive the contacting force using a voltage controller. VCM voltage can be controlled with a varying frequency pulse width modulation (PWM) controller or a linear mode driver [26, 27], as shown in Figure 6(b). When fractional-order tracker locates the specific coordinate point using (12), then the force feedback, *F*_*j*_′, is estimated as

(16) Fj′Fcosθj,2,θj,2cos−1⁡δ2+β2−γ22δβ=cos−1⁡δγ2+δ2θj,2≈cos−1⁡rj−rj−1γ2+rj−rj−12,

where constant force, *F*, is the slide forward or backward motion, force, *F*_*j*_′, is the mock friction between the catheter and the vessel wall, *j* = 1,2, 3,…, *N*_*F*_, where *N*_*F*_ is the number of specific points, and *r*_*j*_ *∠θ*_*j*_ is the polar coordinate of specific point, as seen in Figure 4(b); *γ* is the radius of the blood vessel and *α* ≈ (*r*_*j*_ − *r*_*j*−1_). The VCM's input voltages are proportional to the friction, which can be represented as

(17) $$\begin{array}{c} \frac{F_{j}^{\prime }}{F}=\frac{V_{out}}{V_{min}}⟹V_{j,out}=V_{min}\times \frac{F_{j}^{\prime }}{F}=\frac{V_{min}}{\cos\left(\theta_{j,2}\right)}. \end{array}$$

A buck-boost converter (BBC) [28, 29] is used to regulate the output voltage, *V*_out_, and output current, *I*_out_, by varying the duty cycle of the switching time, *T*_*d*_ (DC/DC). The duty cycle of the voltage control signal is defined as

(18) $$\begin{array}{c} D=\frac{t_{on}}{t_{on}+t_{off}}=\frac{t_{on}}{T_{d}}, \end{array}$$

where *t*_off_ is the turn-off time and *t*_on_ is the turn-on time, as shown in Figure 6(b). The converter's duty cycle can be estimated, *D*_buck_, for the buck mode and the duty cycle, *D*_boost_, for the boost mode, as [28, 29]

**Figure 7:**
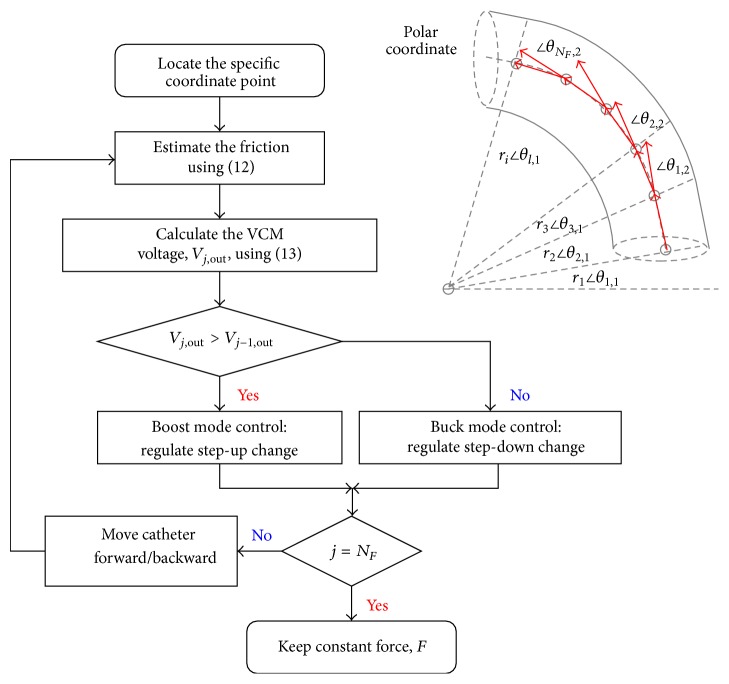
The flowchart of haptic force realization.

(19) DbuckVoutVin<1,

(20) Dboost1−VinVout<1,

where *V*_out_ is the desired output voltage, *V*_out,min_ < *V*_out_ < *V*_out,max_, and *V*_in_ is the input voltage for buck mode and boost mode, *V*_in,min_ < *V*_in_ < *V*_in,max_. The duty cycle is always positive and less than 1. The voltage regulates step-up or step-down changes using

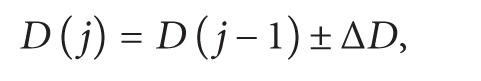
(21)

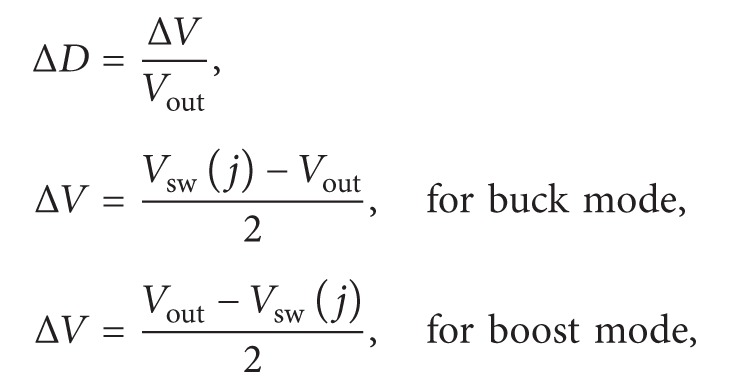
(22)
where *j* = 1,2, 3,…, *N*_*F*_ is the switching number; *V*_out_ is the desired voltage, which is estimated using (17); *V*_sw_(*j*) is the instantaneous voltage at each switching step and its value slowly rises or falls. The flowchart of haptic force realization is shown in Figure 7.

## 4. Experimental Results

### 4.1. VR Mechanism Establishment

**Figure 8:**
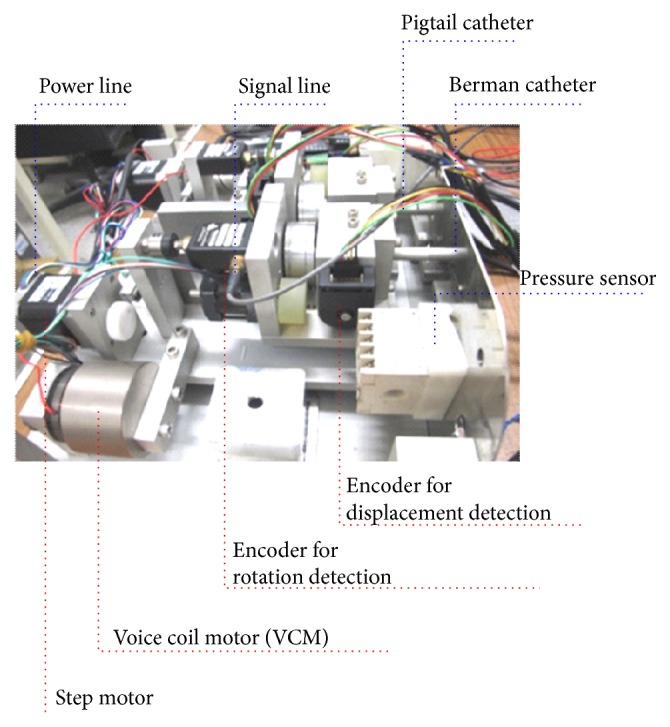
The double-catheter mechanism for ICC.

**Figure 9:**
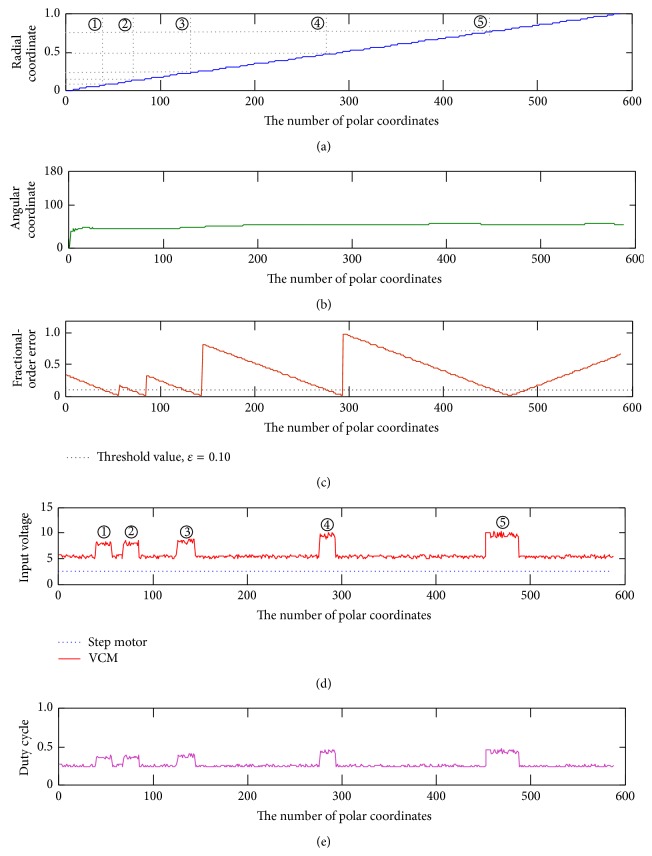
(a) Radial coordinates of the blood vessel and tortuous sites (*①* → *⑤*), (b) angular coordinates of the blood vessel, (c) tracking evaluations with the fractional-order errors, (d) varying input voltages with the PWM control, and (e) varying duty cycles with the buck-boost control.

The proposed VR simulator system for double ICC was set up in a laboratory. The hardware mechanism of the double-catheter system includes two VCMs, two step motors, and four encoders, as shown in Figure 8. The friction forces and haptic forces were realized by two step motors and two VCMs, which were used to mock the double catheters from the right femoral vein into the ventricular and left femoral artery through the right ventricle, respectively. Four encoders were employed to detect the catheter's displacements and rotations, respectively. An encoder for displacement detection provided the displacement information to produce visual feedback, and another encoder for rotation detection synchronously transmitted control signals to trigger friction feedback. Constant friction force was produced by a step motor with feeding fixed input voltage, 2.5 VDC, as shown in Figure 9(d). Visual feedback unit displayed the scenario films during pulling or pushing of a catheter (moving frequency 0.1 Hz to 100 Hz for the operation [7]). Using the friction and visual promoting information, the operator can sense the hand feeling and know the real position of the human body.

Two VCMs were used to produce the haptic forces. Using PWM, (19) to (22) were used to regulate the input voltage of a VCM. This was accomplished by using a fixed switching frequency and varying the duty cycles. The surgeon console worked well between 1 Hz and 10 Hz [7]. A PWM performed on/off switching with a frequency of 10 Hz/s. This also results in less power loss in the switching devices. In addition, the proposed fractional-order vascular access tracker was used to locate the tortuous sites along the vessel trajectory and then switched duty cycles to drive a VCM; the haptic force producer varied input voltages to mock vascular access resistances, “force (*F*)∝ electrical potential (*V*),” between the catheter and the blood vessel wall.

A control (analog-to-digital, USB-6363, NI) card was applied to obtain information or to transmit control signals between the compatible PC and hardware mechanism. A VR simulator system combining haptic force producer, software controller, and image processing unit for ICC can be established using LabVIEW programming (National Instruments Corporation, Austin, Texas, USA) and MATLAB software (MathWorks, Natick, Massachusetts). The feasibility tests will be used to validate the proposed VR system, as detailed below.

### 4.2. Tracking Performance for Locating Tortuous Sites

As shown in Figure 3, there were five vascular tortuous sites, as depicted by the symbol “⊗.” We had prespecific sites to train young surgeons or PGY students. Their Cartesian coordinates and polar coordinates (radial and angular coordinates) are shown in Figures 3, 9(a), and 9(b), respectively. The operators need to ensure and enhance the safety during ICC. The blood vessel will be easily broken at these specific sites. In such cases, the proposed VR system could be used to provide the senior surgeons' skills and experiences to train the unskilled surgeons. We implemented the VR experiment with the surgeons' consoled vessel trajectories.

The proposed fractional-order vascular access tracker was used to track and locate the specific sites, while the fractional-order error, Ψ < *ε* = 0.10, means that the catheter tip arrives at the specific coordinates.  Figure 9(c) shows the radial coordinate tracking results between the surgeons' consoled vessel trajectory and the actual operated trajectory. Then, haptic force producer was used to drive the VCMs and control the visual feedback at each switching cycle with a frequency of 10 Hz/s. The experimental results showed that the proposed tracker could match the surgeons' consoled vessel trajectories.

### 4.3. Haptic Performance at Blood Vessel Trajectory

Based on the friction feedback to an operator's hand and the visual feedback on the monitoring screen, the operator can decide whether to forward/backward the catheter or not. A VCM was used to mock the elasticity of the vessel wall, and a PWM performed on/off switching with a frequency of 10 Hz/s to achieve consoled hand feeling. Electrical mechanism produced fixed resistances at axial direction and elasticity at tortuous sites. The resistance is proportional to the electrical potential, which is *F* ∝ *V*. Haptic forces are also correlated with natural resistances and various angles as depicted in (17). Therefore, the average duty cycle, *D*_ave_ = 0.2511, controlled the BBC to regulate the average input voltage, *V*_ave_ = 5.3542 VDC, at the straightforward vascular access, *D*_ave_ = 0.4122, to regulate *V*_ave_ = 7.7951 VDC (angle: 45°–50°) at tortuous sites, *①* to *③*, and *D*_ave_ = 0.4627 to regulate *V*_ave_ = 9.7202 VDC (angle: 55°–60°) at tortuous sites, *④* to *⑤*, using (19) to (22), respectively. The overall results of input voltages and duty cycles are shown in Figures 9(d) and 9(e). These experimental results showed that the forces appear in a nonlinear behavior in specific regions of the operation. The proposed haptic force producer could provide VR applications in practical procedure.

## 5. Conclusion

In this study, a double-catheter VR simulator system has been established for ICC, which provided some functions to (1) mock the Berman and pigtail catheters on both sides for young surgeons or PGY students to synchronously operate in real-time applications, (2) use the vascular access tracker to follow the senior surgeons' consoled trajectory, in order to learn skills and experiences at the specific sites, and (3) provide the haptic force, friction force, and visual feedback, which established the consoled hand feeling and displayed the frontal and lateral X-ray images to observe the precise position of the human body. In addition, senior surgeons can assign the specific sites to train unskilled surgeons, such as advancing through the tortuous vessels, aortic arch, or vein into the ventricle. The operators could also know the moving frequency and the time consumed for each ICC procedure.

The proposed VR system achieved simulating the ICC procedure using a mechanical design and control. To practice the double-catheter ICC procedures, we have basic and intermediate skills to operate double catheters, such as spatiotemporal location, hand-eye coordinate, and two-handed operation at the straightforward and tortuous vascular accesses, including coronary artery, right femoral vein/left femoral artery, and carotid arteries. Advanced skills can be also applied for further applications, including therapies for peripheral vascular disease (PVD), ASD, endovascular graft-stent placement, and neurosurgery. For example, the proposed VR system could be used to mock PVD treatments to reconstruct occluded vessel access or reposition stents. It needs 3D imaging guidance to track vascular structures and force control to perform surgical repairs. Thus, 3D image servo-control and catheter tip sensor can enhance the visual feedback, which can provide the catheter's motion trajectories and the specific sites in the human body.

In addition, promising therapy for treating vascular diseases is interventional radiology; hence a guidewire-catheter combination and fluoroscopic guidance carry out more localized therapy to reduce recovery time for the patient when compared to traditional surgical procedures [30]. VR system also mocks contrast medium injections and simulates contrast medium washout in the vessel injected operations. The operators can choose the contrast injection volume and the rate relative to the designated blood flow in vascular interventional surgery [31]. It is used to make sure of the anatomic locations and to enhance the visibility of blood vessels. Physiological measurements, such as blood pressure (BP), mean arterial pressure (MAP), heart rate variability (HRV), respiration rate (RR), and cardiac output (CO), can provide important indicator to virtual presentation, while operators choose different scenarios in clinical practices. The hemodynamic characteristics describe simulated patient physiology during any given cardiac cycle, such as heart rate, vascular compliance, vessel resistance, and aortic root pressure [31, 32]. We also expect that the proposed VR simulator system can be applied in surgery education, angioplasty, stenting, catheter-based drug delivery, and clinical training usages; the training purposes can further consider senior surgeons' experiences to make the young surgeons or PGY students highly improve their skills after a series of training and practices.
